# Effects of treating helminths during pregnancy and early childhood on risk of allergy‐related outcomes: Follow‐up of a randomized controlled trial

**DOI:** 10.1111/pai.12804

**Published:** 2017-10-05

**Authors:** Benigna Namara, Stephen Nash, Swaib A. Lule, Hellen Akurut, Harriet Mpairwe, Florence Akello, Josephine Tumusiime, Moses Kizza, Joyce Kabagenyi, Gyaviira Nkurunungi, Lawrence Muhangi, Emily L. Webb, Moses Muwanga, Alison M. Elliott

**Affiliations:** ^1^ MRC/UVRI Uganda Research Unit Entebbe Uganda; ^2^ London School of Hygiene and Tropical Medicine London UK; ^3^ Entebbe Hospital Entebbe Uganda

**Keywords:** albendazole, asthma, atopy, eczema, helminth, IgE, praziquantel, prenatal, school age, wheeze

## Abstract

**Background:**

Helminth infections, common in low‐income countries, may protect against allergy‐related disease. Early exposure may be a key. In the Entebbe Mother and Baby Study, treating helminths during pregnancy resulted in increased eczema rates in early childhood. We followed the cohort to determine whether this translated to increased asthma rates at school age.

**Methods:**

This randomized, double‐blind, placebo‐controlled trial, conducted in Entebbe, Uganda, had three interventions. During pregnancy, women were randomized, simultaneously, to albendazole vs placebo and to praziquantel vs placebo. Their children were independently randomized to quarterly albendazole vs placebo from age 15 months to 5 years. We here report follow‐up to age 9 years. Primary outcomes at 9 years were recent reported wheeze, skin prick test positivity (SPT) to common allergens and allergen‐specific IgE positivity to dust mite or cockroach. Secondary outcomes were doctor‐diagnosed asthma and eczema rates between 5 and 9 years, recent eczema, rhinitis and urticaria at 9 years, and SPT and IgE responses to individual allergens.

**Results:**

2507 pregnant women were enrolled; 1215 children were seen at age nine, of whom 1188 are included in this analysis. Reported wheeze was rare at 9 years (3.7%) while SPT positivity (25.0%) and IgE positivity (44.1%) were common. There was no evidence of a treatment effect for any of the three interventions on any of the primary outcomes.

**Conclusions:**

Prenatal and early‐life treatment of helminths, in the absence of change in other exposures, is unlikely to increase the risk of atopic diseases later in childhood in this tropical, low‐income setting.

## Graphical abstract

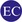

## 1 INTRODUCTION

Allergy‐related diseases (ARD) such as asthma, eczema and rhinitis are commoner in high‐income countries (HICs) than in low‐ or middle‐income countries (LMICs), but their prevalence is increasing in the latter, where consequences can be more severe because of limited access to diagnosis, care and necessary treatment.^1^

Understanding exposures that “protect” against ARD in LMICs is important—for public health, and to provide insights into possible interventions for those at risk of ARD. Helminth infections are highly prevalent in LMICs, and immune responses to helminths and allergens are closely related. IgE and related effector mechanisms are likely to have evolved to protect mammals from helminths and ectoparasites,^2^ but parasitic helminths must survive for decades in their hosts, and have evolved a myriad of mechanisms to evade or suppress the host immune response.^3^ Animal models provide strong evidence that these mechanisms can protect the host against allergy‐related outcomes.^4^

In humans, the picture is more complex. Despite inverse associations between helminths and atopy, several trials have shown no, or little, effect of anthelmintic treatment on allergy‐related outcomes.^4^ One possible explanation is that early‐helminth exposure has long‐term effects, not mitigated by later treatment. In the Entebbe Mother and Baby Study (EMaBS), a randomized, placebo‐controlled trial of anthelmintic treatment during pregnancy, we showed a striking increase in the incidence of infantile eczema following maternal treatment with albendazole in a population with a high prevalence of maternal hookworm infection. We also observed an increase in eczema among infants of mothers with schistosomiasis who were treated with praziquantel.^5^ This was hailed as the first demonstration that treatment during pregnancy influenced ARD in the offspring.^6^

As eczema in early childhood may herald the development of asthma at school age, and as eczema and asthma may have a common link to atopy,^7^ we followed the EMaBS cohort to find out whether the prenatal intervention influenced risk of asthma and other allergy‐related diseases at age 9 years.

## 2 METHODS

### 2.1 Study design and participants

The Entebbe Mother and Baby Study (EMaBS) was a factorial randomized, placebo‐controlled trial of anthelmintic treatment during pregnancy and early childhood conducted in Entebbe, Uganda—a semi‐urban setting in equatorial East Africa [ISRCTN32849447].^8^, ^9^ Healthy pregnant women resident in the study area and planning to deliver at Entebbe Hospital, with no evidence of helminth‐related pathology, were recruited between 2003 and 2005 and randomized to two interventions simultaneously^1^ to receive single‐dose albendazole (400 mg) or matching placebo and^2^ to single‐dose praziquantel (40 mg/kg) or matching placebo, with all treatments received during the second or third trimester of pregnancy. When the offspring turned 15 months, they were randomized independently from the maternal randomization to a third intervention—quarterly albendazole (200 mg below 2 years of age, 400 mg thereafter) or matching placebo—which they received until they turned 5 years, making this a (2 × 2)×2 factorial design. The cohort has continued under follow‐up after completion of the trial interventions. We now report on the evaluation of allergy‐related clinical outcomes from age 5 to 9 years and on prevalence of atopy and allergy‐related disease at 9 years, undertaken to assess longer term impact of early‐life interventions. Participants, clinicians and laboratory staff remain blinded to treatment allocation; only the trial statisticians have access to the randomization code.

The study was approved by the Research and Ethics Committee of the Uganda Virus Research Institute, the Uganda National Council for Science and Technology and the London School of Hygiene & Tropical Medicine.

### 2.2 Study procedures

Children were reviewed by trained health care providers at the research clinic at scheduled annual visits for clinical information and stool examination for helminth infections. Children were additionally seen when they were sick, and all illness events recorded. At age nine, each child was assessed for allergy‐related conditions and atopy by history, examination and skin prick testing (SPT). A blood sample was taken for immunological studies, including evaluation of allergen‐specific immunoglobulin E (asIgE).

Stool samples were examined by the Kato‐Katz method for intestinal helminths: two slides from a single sample were examined at each annual visit.

All study procedures were carried out by healthcare staff trained in the respective fields and guided by standard operating procedures.

### 2.3 Outcomes

Recent reported wheeze, eczema and rhinitis were ascertained using the International Study on Allergy and Asthma in Children (ISAAC) questionnaire,^10^ with supplementary questions for urticaria. Reported wheeze and eczema (a recurrent itchy rash with typical flexural distribution) were classified according to responses from mothers on behalf of their children; “recent” was defined as within 12 months.

Visible flexural dermatitis was defined as described by Williams et al, and all clinicians were trained using the available online tool.^11^

Doctor‐diagnosed asthma and eczema were established by doctors or clinical officers at either routine or illness visits.

Atopy (SPT): SPT was performed using standard procedures.^12^ Allergens tested were *Dermatophagoides*,* Blomia tropicalis*, German cockroach, cat, mould, grass pollen, Bermuda grass and peanut (ALK‐Abelló, Laboratory Specialities (Pty) Ltd, Randburg, South Africa). A test was classified as positive for an allergen if there is a papule of average size >3 mm (while the saline negative control was negative) and negative if there is no papule or a papule of average size <3 mm (while the histamine positive control was positive).

Atopy (Allergen‐specific IgE *specific to Dermatophagoides mixture and German cockroach [Blatella germanica]**)*** was measured as previously described.^5^ Samples were considered positive if results were above the limit of detection, 312.5 ng/mL.

Forced expiratory volume in one‐second (FEV_1_) was measured using a hand‐held spirometer (Micro 1 Diagnostic Spirometer, CareFusion, Chatham Marine, UK). The best result of three forced expirations was recorded.

### 2.4 Statistical considerations and analysis

The analysis aimed to determine the impact of each treatment (maternal albendazole, maternal praziquantel, infant albendazole) on allergy‐related outcomes between age 5 and 9 years. Based on our factorial study design, our primary analysis for each of these three treatments was “everyone who received a particular treatment” vs “everyone who did not receive that treatment.” All children who attended at 9 years were included in the analysis with the exception of children from multiple births, in which case just the first‐born child was included. Additionally, children who attended previous annual visits or were seen by a doctor between the ages of five and nine were included in the analysis of rates of asthma and eczema.

The primary outcomes were reported wheeze in the last 12 months, SPT positivity to one or more allergens, and detectable asIgE at 9 years. We also analysed individual SPT results, recent reported rhinitis and urticaria at 9 years of age and doctor‐diagnosed rates of asthma and eczema between the ages of five and nine. We included each reported asthma and eczema diagnosis, with the exception of diagnoses which occurred with 2 weeks of each other, which were considered the same event and were recorded with the earliest date.

We expected that 1000 children would be seen at 9 years with reported wheeze 10%‐15%, and SPT positivity and IgE prevalence approximately 30%, giving 80% power with *P* < .05 to detect a difference between trial arms of 6% in the proportion of children with wheeze and 9% in the proportion with positive SPT or positive asIgE.

All outcomes were assessed using regression models (logistic for binary outcomes, linear for continuous measurements, Poisson for rates), which included all randomized treatments but no other factors. Confounding with maternal hookworm and schistosomiasis was examined for primary outcomes. The Poisson model included gamma distributed random effects to account for clustering of allergy events by infant. Additional, pre‐planned, analyses were performed in two subgroups. We investigated the effect of maternal albendazole on children of mothers with and without hookworm and the effect of maternal praziquantel on children of mothers with and without schistosomiasis. These analyses were carried out by introducing an interaction term between variables representing the randomized treatment and the worm infection in each regression model described above. To account for multiple comparisons, 99% confidence intervals are reported throughout.

## 3 RESULTS

**Figure 1:**
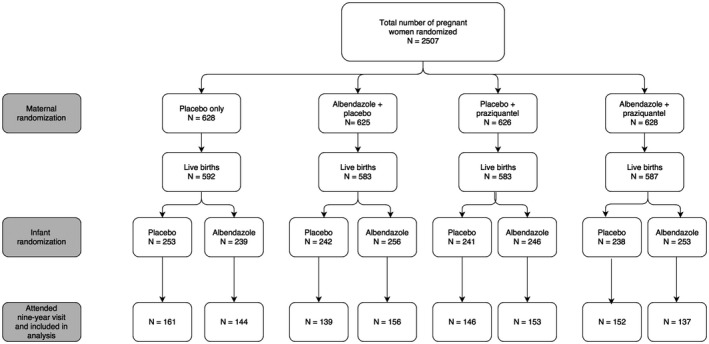
CONSORT flow diagram

A total of 2507 pregnant women were recruited. This analysis includes only children seen at the 9‐year‐old annual visit. Excluding 13 second‐born twins and 13 children who were not randomized to the intervention in childhood, we had data on 1188 (47.2%) mother‐child pairs who had received all three randomized treatments (Figure 1). Compared to mothers whose children did not attend the 9‐year visit, mothers of children included in this article were older (mean age 24.4 vs 23.0 years), more likely to be Baganda (the tribe traditionally based in central Uganda; 55.2% vs 45.4%), multiparous (30.4% vs 22.6%) and to belong to a household with high social‐economic status (58.0% vs 49.5% in class 4 or higher on a six‐point scale).

Due to the (2 × 2)×2 structure of the trial, there were eight possible treatment combinations. The most notable difference in baseline characteristics was in maternal hookworm infection within the infant albendazole randomization (38.9% in active treatment vs 46.1% in placebo; Table 1).

**Table 1:** Baseline characteristics

Characteristic	Total (n = 1188)	Mother received albendazole (n = 584)	Mother received placebo (n = 604)	Mother received praziquantel (n = 588)	Mother received placebo (n = 600)	Child received albendazole (n = 590)	Child received placebo (n = 598)
Maternal characteristics (prior to interventions given during pregnancy)							
Maternal age group (%)							
14‐19	241 (20.3)	125 (20.7)	116 (19.9)	123 (20.5)	118 (20.1)	121 (20.2)	120 (20.3)
20‐24	443 (37.3)	234 (38.7)	209 (35.8)	237 (39.5)	206 (35.0)	220 (36.8)	223 (37.8)
25‐29	286 (24.1)	134 (22.2)	152 (26.0)	132 (22.0)	154 (26.2)	153 (25.6)	133 (22.5)
30‐34	142 (12.0)	73 (12.1)	69 (11.8)	68 (11.3)	74 (12.6)	71 (11.9)	71 (12.0)
35+	76 (6.4)	38 (6.3)	38 (6.5)	40 (6.7)	36 (6.1)	33 (5.5)	43 (7.3)
Maternal education (%)							
None	27 (2.3)	14 (2.3)	13 (2.2)	14 (2.3)	13 (2.2)	14 (2.3)	13 (2.2)
Primary	593 (50.0)	300 (49.8)	293 (50.2)	299 (49.8)	294 (50.1)	288 (48.2)	305 (51.8)
Senior	455 (38.3)	226 (37.5)	229 (39.2)	239 (39.8)	216 (36.8)	244 (40.8)	211 (35.8)
Tertiary	112 (9.4)	63 (10.4)	49 (8.4)	48 (8.0)	64 (10.9)	52 (8.7)	60 (10.2)
Maternal tribe (%)							
Muganda	656 (55.2)	341 (56.5)	315 (53.9)	327 (54.5)	329 (56.0)	342 (57.2)	314 (53.2)
Munyankole	99 (8.3)	40 (6.6)	59 (10.1)	57 (9.5)	42 (7.1)	49 (8.2)	50 (8.5)
Mutoro	46 (3.9)	28 (4.6)	18 (3.1)	22 (3.7)	24 (4.1)	26 (4.3)	20 (3.4)
Musoga	41 (3.5)	18 (3.0)	23 (3.9)	19 (3.2)	22 (3.7)	17 (2.8)	24 (4.1)
Luo	58 (4.9)	34 (5.6)	24 (4.1)	30 (5.0)	28 (4.8)	25 (4.2)	33 (5.6)
Munyarwanda	54 (4.5)	27 (4.5)	27 (4.6)	28 (4.7)	26 (4.4)	22 (3.7)	32 (5.4)
Other	234 (19.7)	116 (19.2)	118 (20.2)	117 (19.5)	117 (19.9)	117 (19.6)	117 (19.8)
Household socioeconomic status (%)							
1 lowest	70 (6.0)	39 (6.6)	31 (5.4)	35 (5.9)	35 (6.0)	24 (4.1)	46 (7.9)
2	81 (6.9)	38 (6.4)	43 (7.4)	42 (7.1)	39 (6.7)	40 (6.8)	41 (7.1)
3	341 (29.1)	178 (30.0)	163 (28.2)	170 (28.7)	171 (29.5)	192 (32.4)	149 (25.7)
4	348 (29.7)	179 (30.2)	169 (29.2)	173 (29.2)	175 (30.2)	166 (28.0)	182 (31.4)
5	252 (21.5)	121 (20.4)	131 (22.7)	133 (22.5)	119 (20.6)	131 (22.1)	121 (20.9)
6 highest	79 (6.7)	38 (6.4)	41 (7.1)	39 (6.6)	40 (6.9)	39 (6.6)	40 (6.9)
Maternal gravidity (%)							
1	269 (22.6)	127 (21.0)	142 (24.3)	146 (24.3)	123 (20.9)	135 (22.6)	134 (22.7)
2‐4	689 (58.0)	362 (59.9)	327 (56.0)	344 (57.3)	345 (58.7)	350 (58.5)	339 (57.5)
≥5	230 (19.4)	115 (19.0)	115 (19.7)	110 (18.3)	120 (20.4)	113 (18.9)	117 (19.8)
Maternal infections (%)							
Hookworm	503 (42.6)	258 (42.9)	245 (42.2)	244 (40.8)	259 (44.3)	274 (46.1)	229 (38.9)
Schistosoma mansoni	228 (19.3)	121 (20.1)	107 (18.4)	108 (18.1)	120 (20.5)	117 (19.7)	111 (18.9)
Malaria parasites	107 (9.2)	59 (9.9)	48 (8.4)	64 (10.9)	43 (7.5)	63 (10.7)	44 (7.6)
HIV positive	114 (9.6)	56 (9.3)	58 (9.9)	62 (10.3)	52 (8.8)	58 (9.7)	56 (9.5)
Child characteristics (prior to intervention between 15mo and 5y)							
Infant birthweight (205 mv)	3.19 (0.48)	3.21 (0.48)	3.18 (0.48)	3.19 (0.48)	3.20 (0.48)	3.20 (0.45)	3.19 (0.51)
Child sex (%)							
Male	612 (51.5)	316 (52.3)	296 (50.7)	317 (52.8)	295 (50.2)	318 (53.2)	294 (49.8)
Female	576 (48.5)	288 (47.7)	288 (49.3)	283 (47.2)	293 (49.8)	280 (46.8)	296 (50.2)
Infant HIV (%)							
No	1135 (98.5)	577 (98.5)	558 (98.6)	574 (98.5)	561 (98.6)	568 (98.3)	567 (98.8)
Yes	17 (1.5)	9 (1.5)	8 (1.4)	9 (1.5)	8 (1.4)	10 (1.7)	7 (1.2)

MV, missing values.

At age nine, prevalence of nematodes was slightly lower in children who had been treated with albendazole quarterly to age five, but the prevalence of helminths was low, with the exception of schistosomiasis (Table 2).

**Table 2:** Worm prevalence at 9‐year visit by infant randomization group

Species	Placebo (n = 597)^a^ (%)	Albendazole (n = 589)^a^ (%)
Trichuris	24 (4.02)	21 (3.57)
Ascaris	6 (1.01)	2 (0.34)
Schistosomiasis	64 (10.72)	72 (12.22)
Hookworm	6 (1.01)	0
Trichostrongylus	0	0
Other	13 (2.18)^b^	7 (1.19)^c^

a Stool samples were unavailable for one infant in each randomization group.

b Other species were *Hymenolepis nana* (10 cases), *Enterobius vermicularis*.^3^

c Other species were *Hymenolepis nana,* 5 *Enterobius vermicularis*.^3^ One child was infected with both species.

### 3.1 Primary outcomes

At age 9 years, data were available on recent reported wheeze for 1143 (96.2%) children, SPT allergen response for 1140 (96.0%) and allergen‐specific IgE response for 1096 (92.3%). The overall prevalence of recent reported wheeze was 3.85%, of SPT positivity 25.0% and of detectable IgE to house dust mite, cockroach or both 44.1%. There was no evidence of an effect of randomized treatment on any primary outcome (Table 3). There was no evidence of confounding or of any interaction between treatments (*P* > .1 for all interactions).

**Table 3:** Primary outcomes by randomization group

Outcome	Maternal randomizations				Infant randomization	
	Placebo	Albendazole	Placebo	Praziquantel	Placebo	Albendazole
Wheeze (last 12 mo) Odds ratio (99% CI)	26/580 (4.5%)	18/563 (3.2%)	18/583 (3.1%)	26/560 (4.6%)	22/572 (3.8%)	22/571 (3.9%)
	0.70 (0.31‐1.57)		1.53 (0.69‐3.43)		1.01 (0.46‐2.23)	
Positive for any SPT^a^ Odds ratio (99% CI)	146/576 (25.3%)	139/564 (24.6%)	138/578 (23.9%)	147/562 (26.2%)	144/577 (25.0%)	141/563 (25.0%)
	0.96 (0.68‐1.37)		1.13 (0.79‐1.61)		1.00 (0.71‐1.43)	
Allergen‐specific IgE positive to dust or cockroach Odds ratio (99% CI)	239/557 (42.9%)	244/539 (45.3%)	233/556 (41.9%)	250/540 (46.3%)	242/559 (43.3%)	241/537 (44.9%)
	1.10 (0.80‐1.51)		1.20 (0.87‐1.64)		1.07 (0.78‐1.46)	

a Any of Blomia tropicalis, Dermatophagoides, cockroach, mould, pollens, Bermuda grass, peanut.

In pre‐planned subgroup analyses, we also found no evidence of an interaction between maternal hookworm and maternal albendazole nor maternal schistosomiasis and praziquantel (Table 4).

**Table 4:** Outcomes by selected maternal randomization group in maternal infection subgroups^a^

	Women with hookworm infection		Women without hookworm infection		*P* ‐value^b^	Women with schistosomiasis		Women without schistosomiasis		*P* ‐value^b^
	Placebo	Albendazole	Placebo	Albendazole		Placebo	Praziquantel	Placebo	Praziquantel	
Wheeze (last 12 mo)	7/248 (2.8%)	5/236 (2.1%)	19/329 (5.8%)	13/324 (4.0%)	.89	5/106 (4.7%)	6/115 (5.2%)	13/475 (2.7%)	20/441 (4.5%)	.57
OR (99% CI)^c^	0.75 (0.16, 3.43)		0.68 (0.26, 1.75)			1.12 (0.23, 5.59)		1.69 (0.66, 4.31)		
Positive SPT^d^ for any allergen	58/245 (23.7%)	51/237 (21.5%)	87/328 (26.5%)	87/324 (26.9%)	.63	30/106 (28.3%)	37/112 (33.0%)	106/470 (22.6%)	110/446 (24.7%)	.75
OR (99% CI)^c^	0.89 (0.51, 1.55)		1.01 (0.64, 1.60)			1.25 (0.59, 2.68)		1.12 (0.75, 1.68)		
Allergen‐specific IgE (any positive)	109/241 (45.2%)	106/223 (47.5%)	128/313 (40.9%)	137/313 (43.8%)	.92	46/101 (45.5%)	58/113 (51.3%)	186/453 (41.1%)	190/423 (44.9%)	.84
OR (99% CI)^c^	1.10 (0.68, 1.77)		1.13 (0.74, 1.71)			1.25 (0.62, 2.54)		1.18 (0.83, 1.67)		

a This analysis excludes six mother‐child pairs for whom no maternal stool sample was available for assessment of worm infection. Of these excluded pairs, two children had a positive SPT result, and three had detectable IgE to house dust mite or cockroach.

b *P* ‐value for interaction.

c ORs adjusted for all three randomized treatments.

d Any of Blomia tropicalis, Dermatophagoides, German cockroach, cat, mould, pollens, Bermuda grass, peanut.

### 3.2 Secondary outcomes

We had data on doctor‐diagnosed illness events between ages five and nine for 1679 children. Twenty of these experienced a total of 50 doctor‐diagnosed asthma events, a rate of 8.5 per 1000 years of follow‐up, giving limited statistical power. There was no clear evidence of any effect of treatment on this outcome.

FEV1 measurements were available for 1007 infants (84.8%). The mean FEV1 was 1.41 mL (SD = 0.25), with a range of 0.68‐2.5. There was no evidence of an effect of treatment on FEV_1_ (Table 5).

**Table 5:** Secondary outcomes in different randomization groups

Outcome^a^	Maternal randomizations				Infant randomization	
	Placebo	Albendazole	Placebo	Praziquantel	Placebo	Albendazole
Doctor‐diagnosed asthma; events (individuals)	41 (14)	9 (6)	24 (9)	26 (11)	33 (12)	17 (8)
Hazard ratio (99% CI)	0.26 (0.05‐1.31)		1.01 (0.20‐4.98)		0.54 (0.11‐2.69)	
FEV1; mean (SD; n)	1.42 (0.24; 509)	1.40 (0.25; 498)	1.41 (0.24; 513)	1.40 (0.25; 494)	1.41 (0.25; 511)	1.41 (0.25; 496)
Mean difference (99% CI)	−0.02 (−0.06, 0.02)		−0.01 (−0.05, 0.03)		0.00 (−0.04, 0.04)	
Doctor‐diagnosed eczema; events (individuals)	30 (13)	35 (25)	29 (22)	36 (16)	23 (20)	42 (18)
Hazard ratio (99% CI)	1.25 (0.44‐3.59)		1.27 (0.43‐3.74)		1.78 (0.61‐5.20)	
Recent reported eczema (%)	20/604 (3.31)	38/584 (6.51)	25/600 (4.17)	33/588 (5.61)	29/598 (4.85)	29/590 (4.92)
Odds ratio (99% CI)	2.03 (0.98, 4.21)		1.37 (0.68, 2.77)		1.01 (0.51, 2.03)	
Visible flexural eczema at 9 y (%)	10/604 (1.66)	13/584 (2.23)	9/600 (1.50)	14/588 (2.38)	11/598 (1.84)	12/590 (2.03)
Odds ratio (99% CI)	1.35 (0.45, 4.04)		1.60 (0.53, 4.87)		1.11 (0.38, 3.30)	
Recent reported rhinitis (%)	28/564 (5.0)	24/552 (4.3)	17/563 (3.0)	35/553 (6.3)	26/563 (4.6)	26/553 (4.7)
Odds ratio (99% CI)	0.87 (0.42‐1.82)		2.17 (1.00‐4.72)		1.03 (0.49‐2.15)	
Recent reported urticaria (%)	88/583 (15.1)	88/558 (15.8)	88/578 (15.2)	88/563 (15.6)	93/574 (16.2)	83/567 (14.6)
Odds ratio (99% CI)	1.05 (0.69‐1.61)		1.03 (0.68‐1.57)		0.89 (0.58‐1.35)	
Dermatophagoides SPT positive (%)	103/573 (18.0)	104/559 (18.6)	109/571 (19.1)	98/561 (17.5)	105/573 (18.3)	102/559 (18.2)
Odds ratio (99% CI)	1.04 (0.70‐1.55)		0.90 (0.60‐1.33)		0.99 (0.67‐1.48)	
Blomia tropicalis SPT positive (%)	90/570 (15.8)	83/556 (14.9)	80/566 (14.1)	93/560 (16.6)	87/571 (15.2)	86/555 (15.5)
Odds ratio (99% CI)	0.93 (0.61‐1.43)		1.21 (0.79‐1.86)		1.02 (0.67‐1.56)	
German cockroach SPT positive (%)	61/573 (10.6)	62/558 (11.1)	57/569 (10.0)	66/562 (11.7)	65/572 (11.4)	58/559 (10.4)
Odds ratio (99% CI)	1.05 (0.64‐1.71)		1.19 (0.73‐1.96)		0.90 (0.55‐1.48)	
Cat SPT positive (%)	7/570 (1.2)	4/556 (0.7)	3/567 (0.5)	8/559 (1.4)	3/568 (0.5)	8/558 (1.4)
Odds ratio (99% CI)	0.58 (0.11‐2.97)		2.72 (0.47‐15.69)		2.71 (0.47‐15.64)	
Mould SPT positive (%)	2/571 (0.4)	1/555 (0.2)	0/565 (0.0)	3/561 (0.5)	0/570 (0.0)	3/556 (0.5)
Odds ratio (99% CI)	0.54 (0.02‐12.90)		–		–	
Pollens SPT positive (%)	4/572 (0.7)	8/556 (1.4)	6/568 (1.1)	6/560 (1.1)	5/571 (0.9)	7/557 (1.3
Odds ratio (99% CI)	2.07 (0.42‐10.10)		1.01 (0.23‐4.53)		1.44 (0.32‐6.55)	
Bermuda grass SPT positive (%)	7/573 (1.2)	7/557 (1.3)	7/568 (1.2)	7/562 (1.2)	4/573 (0.7)	10/557 (1.8
Odds ratio (99% CI)	1.02 (0.26‐4.10)		1.01 (0.25‐4.05)		2.60 (0.56‐12.03)	
Peanut SPT positive (%)	6/573 (1.0)	9/557 (1.6)	7/568 (1.2)	8/562 (1.4)	5/572 (0.9)	10/558 (1.8)
Odds ratio (99% CI)	1.55 (0.39‐6.09)		1.16 (0.30‐4.44)		2.07 (0.50‐8.56)	
House Dust Mite IgE positive (%)	159/557 (28.5)	165/539 (30.6)	158/556 (28.4)	166/540 (30.7)	161/559 (28.8)	163/537 (30.4)
Odds ratio (99% CI)	1.10 (0.78‐1.55)		1.12 (0.80‐1.58)		1.08 (0.77‐1.52)	
Cockroach IgE positive (%)	173/557 (31.1)	182/539 (33.8)	170/556 (30.6)	185/540 (34.3)	176/559 (31.5)	179/537 (33.3)
Odds ratio (99% CI)	1.13 (0.81‐1.58)		1.19 (0.85‐1.66)		1.09 (0.78‐1.52)	

a The results given are number of children with the characteristic/number tested (%) unless otherwise specified.

Thirty‐eight infants experienced a total of 65 doctor‐diagnosed eczema events, a rate of 11.0 per 1000 years. There were 58 infants with reported eczema in the 12 months prior to their 9‐year assessment, but only 29 cases of flexural dermatitis (eczema) discovered at examination at 9‐year assessments, six of which were related to skin lesions in the finger webs indicative of scabies burrows and so were not classified as flexural dermatitis according to our protocol. Maternal albendazole was associated with a twofold increase in reported eczema, but the confidence interval was wide (OR 2.03; 99% CI 0.98, 4.21). OR point estimates were >1.00 for effects of maternal and childhood treatment on other measures of eczema, but again confidence intervals were wide.

There were 52 (4.7%) children with recent rhinitis at age nine. There was some evidence of a deleterious effect of maternal praziquantel on rhinitis (OR 2.17; 99% CI 1.00‐4.72), but no evidence of any other effect of randomized treatment on this outcome.

The most common SPT positives were to *Dermatophagoides* (207, 18.3%), *Blomia* (173, 15.4%) and German cockroach (123, 10.9%). There was no evidence of any treatment effect on these SPT responses. There were insufficient positives to other allergens to draw firm conclusions.

## 4 DISCUSSION

This trial is the first to investigate effects of anthelminthic intervention during pregnancy and early childhood on allergy‐related outcomes at school age. We found no statistically significant effect—importantly no adverse effect—of maternal albendazole or praziquantel treatment during pregnancy, or of early‐childhood treatment with albendazole, on the primary outcomes of wheeze in the last 12 months, or SPT response, at age 9 years.

As we have previously reported, our trial interventions during pregnancy had a substantial impact on helminth prevalence among mothers (albendazole treatment reduced hookworm infection (the commonest nematode) to 5%, compared to 45%; praziquantel reduced maternal *S. mansoni* infection to 5% compared with 21%^13^) and there were striking effects of maternal treatment on the incidence of infantile eczema: increases of the order of twofold.^5^ Therefore, we seemed well placed to investigate whether these effects translated to an impact on allergy‐related outcomes at school age. However, we encountered a key limitation in the lower‐than‐expected prevalence of wheeze and incidence of doctor‐diagnosed asthma: recent wheeze was reported by 4% (compared to the predicted 10%‐15%, based on previous studies in Ethiopia and Kenya^14^). No formal testing of airway resistance in response to methacholine was done to contribute to the diagnosis of asthma. Loss to follow‐up made the cohort less representative of the Entebbe population than at the outset, but those retained were of higher SES than those who were lost, perhaps making underestimation of wheeze less likely. Non‐atopic triggers for wheeze may be less common in Entebbe than elsewhere in Africa; for example, moderate temperatures and high humidity are likely to reduce risk of exercise‐induced wheeze.^15^ Education of the cohort on ARD may have resulted in a better understanding of wheeze and asthma than is usual in similar settings, making “false‐positive” reports less likely.

We hypothesized that prenatal and early‐life treatment of helminths might increase the risk of asthma and wheeze. No such effect was observed. If anything, there was a possible reduction among children exposed to prenatal or early‐childhood albendazole. Although weak effects, these accord with findings of Lynch and colleagues^16^ who found that albendazole treatment reduced rates of asthma in a Venezuela cohort. Theoretically, this benefit might represent the effect of a reduced burden of helminths, such as *Ascaris*, which induce wheeze during migration through the lungs, but the low burden of such nematode infections in our cohort makes this explanation unlikely.

Eczema was also uncommon at age nine, as expected with increasing age. Eczema was measured in three ways, all of which gave point estimates for effects of treatment that were greater than one but, again, there was limited statistical power. These results are in keeping with the persistent effect of maternal albendazole treatment observed at age five^17^ and with the observation that eczema in early life was associated with eczema at age 9 years in this cohort (Lule et al, under review, Ped Allergy Immunol). In addition, there was a suggestion of an adverse effect of maternal albendazole treatment on prevalence of rhinitis in 9‐year‐olds. Thus, for ARD other than wheeze, our findings are consistent with a persistent, weak, adverse effect of the early‐life interventions, but are inconclusive.

By contrast, the prevalence of SPT positivity and detectable asIgE was approximately as expected, and hence, we had good power to show that the prenatal and early‐childhood interventions had no effect on atopy at age 9 years. This implies that possible effects of helminths are not mediated by a reduction in the specific IgE response to allergens. Indeed, within this cohort and elsewhere, it has been shown that in the presence of helminths, the link between asIgE and ARD is suppressed^18^ and, similarly, the link between asIgE and histamine release.^19^

Evaluation of the time course of ARD in this cohort showed a high prevalence and incidence of eczema in infancy but a marked decline in ARD with age, and no significant emergence of asthma at school age.^20^ By contrast, the prevalence of atopy increased. This further emphasizes the dissociation between atopy and ARD. Given the modest prevalence of helminth infection to age 9 years in this semi‐urban cohort, we postulate that other exposures also contribute to protection against allergy‐related disease in this environment. Prenatal and early‐life intervention against helminths, in the absence of change in other exposures, is not likely to have any immediate adverse impact on allergy‐related disease risk in tropical, low‐income settings.
